# Competition: A Missing Component of Fruit Fly (Diptera: Tephritidae) Risk Assessment and Planning

**DOI:** 10.3390/insects13111065

**Published:** 2022-11-17

**Authors:** Anthony R. Clarke, Penelope F. Measham

**Affiliations:** 1 School of Biology and Environmental Science, Queensland University of Technology (QUT), GPO Box 2434, Brisbane, QLD 4001, Australia; 2 Horticulture and Forestry Science, Department of Agriculture and Fisheries, GPO Box 267, Ecosciences Precinct Dutton Park, Brisbane, QLD 4102, Australia

**Keywords:** Tephritidae, fruit fly, invasion, competition, pest risk analysis, risk assessment

## Abstract

**Simple Summary:**

Tephritid fruit flies are highly invasive, globally important insect pests of horticulture. Significant effort goes into assessing and managing the risks posed by fruit flies, while substantial investments are made into their control. An important factor in the biology of fruit flies is competition. Different species of fruit fly compete over access to fruit, with more-competitive species able to displace less-competitive species in time, space and host usage. While this behaviour is very well documented scientifically, it is rarely incorporated into pest risk assessment or pest management investment. Targeting regulators and decision makers, this paper reviews the science underpinning our knowledge of fruit fly competition, and then identifies four major effects of fruit fly competition that could impact pest risk planning or large-scale pest management initiative. These are: (i) numerical reduction of an existing fruit fly pest following competition with an invasive fruit fly; (ii) displacement of a less competitive fruit fly pest species in space, time or host; (iii) ecological resistance to fruit fly invasion in regions already with competitively dominant fruit fly species; and (iv) lesser-pest fruit fly resurgence following control of a competitively superior species.

**Abstract:**

Tephritid fruit flies are internationally significant pests of horticulture. Because they are also highly invasive and of major quarantine concern, significant effort is placed in developing full or partial pest risk assessments (PRAs) for fruit flies, while large investments can be made for their control. Competition between fruit fly species, driven by the need to access and utilise fruit for larval development, has long been recognised by researchers as a fundamental component of fruit fly biology, but is entirely absent from the fruit fly PRA literature and appears not be considered in major initiative planning. First presenting a summary of the research data which documents fruit fly competition, this paper then identifies four major effects of fruit fly competition that could impact a PRA or large-scale initiative: (i) numerical reduction of an existing fruit fly pest species following competitive displacement by an invasive fruit fly; (ii) displacement of a less competitive fruit fly pest species in space, time or host; (iii) ecological resistance to fruit fly invasion in regions already with competitively dominant fruit fly species; and (iv) lesser-pest fruit fly resurgence following control of a competitively superior species. From these four major topics, six more detailed issues are identified, with each of these illustrated by hypothetical, but realistic biosecurity scenarios from Australia/New Zealand and Europe. The scenarios identify that the effects of fruit fly competition might both positively or negatively affect the predicted impacts of an invasive fruit fly or targeted fruit fly control initiative. Competition as a modifier of fruit fly risk needs to be recognised by policy makers and incorporated into fruit fly PRAs and major investment initiatives.

## 1. Introduction

Frugivorous tephritid fruit flies (Diptera: Tephritidae) are internationally significant pests of horticulture as adults lay their eggs into sound fruit on-plant, where the subsequent larval feeding causes fruit loss [1,2]. Additionally, recently infested fruit can appear sound and so can be spread through human carriage [3,4]. Because of the quarantine risk this poses, fruit flies can limit access to new markets [5,6] and more International Standards for Phytosanitary Measures explicitly target this one pest group than any other [7].

Despite quarantine and phytosanitary treatments, fruit fly incursions happen frequently [8,9], which may or may not lead to the permanent establishment of a new pest. In the last two decades the introduction and establishment of Oriental fruit fly (*Bactrocera dorsalis* (Hendel)) in sub-Saharan Africa is the most high profile and damaging fruit fly invasion [10], but this is only one of several examples: peach fruit fly (*Bactrocera zonata* (Saunders)) into the eastern and southern Mediterranean basin, olive fruit fly (*Bactrocera oleae* (Rossi)) into the USA, and Oriental fruit fly into central and northern China are others [11].

Because of their invasion potential, significant preparedness effort is spent carrying out partial or full pest risk assessments (PRAs) for fruit flies [12], where a PRA is the “*evaluation of the probability of the introduction and spread of a pest and the magnitude of the associated potential economic consequences*” [13]. The production of pest risk maps (sensu [14]) is common, most using climate matching approaches to predict the likelihood of establishment, distribution and abundance of an invasive fly [15,16,17]. Such maps can be overlain with estimates of pathways and propagule pressure [4], crop production areas [18], or a mix of these [19], to better estimate risk. Full PRAs for fruit flies become highly sophisticated and include, additional to the likelihood of entry and establishment, predictions of spread rate, crop yield loss and economic impact [20,21]. Unfortunately, even the most exhaustive of such analyses ignore a major issue which may significantly modify the biological likelihood and impact of a fruit fly invasion: that different fruit fly species compete.

Competitive interactions between fly species have been long recognised by fruit fly researchers [22,23,24,25]. Reviewing the literature, Duyck et al. [26] concluded that competition was a key component of fruit fly invasions, noting particularly that there was evidence for a competitive hierarchy among tephritids: *Bactrocera* Macquart species were competitively superior to *Ceratitis* MacLeay species, which were competitively superior to *Anastrepha* Schiner species. Since 2004 there has been significant additional work on tephritid competition (e.g., [27,28,29,30,31,32,33,34,35]) and this literature strongly reinforces, with substantial new data, the earlier conclusions of Duyck and colleagues. However, as judged by its absence from the fruit fly PRA documentation read by us, the implications of fruit fly competition for plant health policy and management planning are largely absent from the literature.

In this paper we raise and discuss the implications of what fruit fly competition means for plant health regulators and policy makers, whether in the context of responding to a new fruit fly invasion, or for the ongoing management of established fruit flies. Invasive species are generally more likely to have greater competitive ability than non-invasive species [36] and are more likely to establish as pests in regions with altered ecosystems (such as crop production systems) without natural enemies or other potential competitors [37,38]. However, this is not always the case and so we argue that competition is relevant in risk assessment. Our review does not provide plant health solutions or recommendations, but simply articulates the numerous ways, positive or negative, direct or indirect, that fruit fly competitive interactions may impact on pest risk assessment and related plant health policy considerations.

The research literature on tephritid competition is well covered in other sources [25,26,32,35,39,40,41] and it is not the purpose of this paper to comprehensively review this material from a biology perspective. Rather, after providing only sufficient biological and theoretical background to provide context, the “flow-on effects” of fruit fly competition for plant health policy are identified, discussed, and then illustrated by hypothetical, but technically possible, scenarios. The use of scenarios, “*plausible and often simplified descriptions of how the future might develop*”, is an important part of pest risk assessment [42].

We note that at all times the introduction of an exotic fruit fly will always be, ultimately, a negative event for local horticulture. By identifying potential indirect benefits from an invasion (Section 3) we are not, in anyway, endorsing a strategy for introducing exotic flies or relaxing quarantine. Rather, we identify potential indirect benefits, as well as negatives, so that more accurate assessments of the total risks associated with an invasion can be made. All except the last of our scenarios are based on an assumption of a newly invasive organism that has spread from the point of entry and established a new permanent population, the final part of the invasion process [43]. Costs of the invasion are likely to be greater at this point than the costs associated with short term eradication efforts [44]. Our last scenario differs from the others by dealing not with a future invasive situation, as would be assessed by a PRA, but rather how competition may impact on plant health outcomes if a competitively dominant species, be that species an endemic or an established exotic, is successfully controlled through a targeted, species-specific mechanism (such as the Sterile Insect Technique).

## 2. Biological Background

### 2.1. Theoretical Framework

Fruit fly competition sits within the evolutionary/ecological theoretical framework of the competitive exclusion principle [45] and the competitive displacement and coexistence principles [46], both derived from the earlier work of Gause [47] who postulated that two species with similar ecologies and resource needs cannot live together in the same place (commonly referred to as Gaussian niche theory, or simply niche theory). In summary, these different theories/principles postulate that competitive interactions between species for a limited resource will ultimately favour one species over another as the dominant species increasingly limits access to the resource through weight of numbers or intrinsic competitive advantage. This will, in turn, drive geographic, temporal, or resource displacement in the less competitive species as, in the absence of such displacement, the less competitive species will be reduced in numbers until local extinction occurs [48,49]. Competition as an ecologically important mechanism is a routine part of research in all biological systems from microbes to higher animals [50,51,52,53], but it is not a routine part of assessing risk.

### 2.2. Competition by Invasive Fruit Flies

Following invasion by a fruit fly species into a new environment, competition between the invasive species and fruit fly species already present in the invaded area is routinely observed [26,32,41]. This is manifested through population reduction of the existing species [29,32,34,54,55,56]; geographic displacement of the local species, for example through climatic niche partitioning by species moving from low altitude areas to high altitude areas [31,40,57]; or by changed host-use, such as using different fruit species [40,58,59,60,61], or even different ripening stages of a host [41]. Such competition driven changes are generically referred to as competitive displacement [62]. Ekesi et al. [29] illustrates the displacement of mango fruit fly (*Ceratitis cosyra* (Walker)) by Oriental fruit fly in Kenyan mangoes over the years 2001 to 2008, a period which covers the before, during and post-establishment phase of the Oriental fruit fly invasion in Kenya [63,64]. Vargas et al. [55] presents very similar, long-term (1998–2009) before-and-after data showing the replacement of Queensland fruit fly (*Bactrocera tryoni* (Froggatt)) and *Bactrocera kirki* (Froggatt) by Oriental fruit fly in Tahiti. These two data sets provide hard field data for the displacement of existing species by an invasive species in commercial crops.

Spatial, temporal or host differentiation that is observed following an invasion appears to be due to existing biological differences between species becoming more apparent in a competitive environment. For example, on La Reunion, the host use patterns of already resident polyphagous fruit fly species significantly changed following the introduction of peach fruit fly [59] and then Oriental fruit fly [40]. However, these changes were only rarely due to entirely new hosts being utilised: rather, some hosts previously used by resident flies were no longer used because of competitive exclusion by the invader, while previously infrequently used hosts were used more. Thus, the differences in host ranges of the resident and invasive species were made more obvious because of the competition for the jointly preferred hosts, which pushed the competitively inferior species to less preferred hosts. The temporal seasonal difference between the mango fruit fly and Oriental fruit fly in African mangoes may be a similar example of competition making innate species-level biological differences more obvious. In Benin mangoes, mango fruit fly and Oriental fruit fly have a stable relationship, with mango fruit fly populations peaking early in the mango season, and Oriental fruit fly in the mid to late season: the populations of both species have only minimal overlap in the mango crop [65]. This could be interpreted as competitive exclusion by Oriental fruit fly of mango fruit fly from across nearly the entire mango fruiting season, but as this pattern was recorded very soon after Oriental fruit fly invasion [66], it is more likely due to the innate dry season/wet season preference of mango fruit fly and Oriental fruit fly, respectively [67], with some competitive displacement at the time of seasonal overlap making the competition effect look more obvious.

The strengths of competitive effects may be modulated by the availability or absence of alternate host plants [55,68], seasonal rainfall and temperature patterns [69], the presence of topographic variation which can lead to climatic niche partitioning [31,57], and sometimes a combination of all three [34]. Competition leading to species extinction does not appear to be a normal outcome in fruit fly systems [26]. While competition leading to extinction has been postulated for the case of Queensland fruit fly replacing Mediterranean fruit fly (*Ceratitis capitata* (Wiedemann)) in eastern Australia [70,71], the evidence for competition being the sole driver of the extinction of Mediterranean fruit fly in eastern Australia is entirely inferential (see Supporting Document S1). It is possible that invasive fruit flies have driven rare endemic fruit fly species on Indian Ocean islands to extinction, but with the data available this interpretation is again recognised as inferential [31,57].

### 2.3. Asymmetrical Competition and Competitive Hierarchy 

Contest competition occurs when one group of individuals directly interact with individuals of a second group for access to a limited resource [72]. When contest competition persistently favours one competing group over another it is known as asymmetrical competition: this type of competition is prevalent between insect species [73]. As identified by Duyck et al. [26], most fruit fly competition falls into this category and, further, a competitive hierarchy exists across genera, with *Bactrocera* species more competitive than *Ceratitis* species, which are more competitive than *Anastrepha* species. Within a genus, species are also more or less competitive than others. Since Duyck et al. [26] more pair-wise species interactions have been documented (e.g., [27,60,74]), and more documentation provided for existing cases (e.g., [28,41]), but the hierarchical pattern has not significantly changed [35], although possible exceptions may occur. In South America, competitive interactions between the invasive *Bactrocera carambolae* (Drew & Hancock) and native *Anastrepha* species appears complex, with fruit sampling data finding evidence both for [75] and against [76] competitive displacement of the latter by the former. In the Sudan *B. zonata* is more abundant than *B. dorsalis* in some but not all districts following the arrival of *B. zonata* after *B. dorsalis*, with the authors acknowledging more time is needed to see how numbers of the two species will settle with respect to the other [33]. With the exception of the Sudan study, the competitive dominance of Oriental fruit fly over other *Bactrocera* and *Ceratitis* species has been re-demonstrated numerous times since 2004 [26], including greatly reducing populations of the other important *Bactrocera* pests *B. tryoni* and *B. zonata*, on the islands of Tahiti [55] and La Reunion [40], respectively.

### 2.4. Mechanisms of Competition

The mechanisms by which one fruit fly species gains a competitive advantage over another broadly fall into two categories: demographic advantage and resource utilisation advantage. In terms of demographic advantage, more competitive fruit flies have been shown to be longer lived, have a longer reproductive period, and invest more in individual offspring (i.e., produce larger eggs) than do less competitive flies [77]. With respect to resource utilisation, females of more competitive species may defend the fruit more aggressively from other ovipositing females [29,41,78], while larvae within the fruit can suppress the size and/or number of competing larvae [23,60,74]. Larval advantage is commonly associated with faster larval growth rate [30]: larvae of competitively superior species are more likely to succeed in scramble competition against larvae of the competitively inferior species by growing faster and being larger [29,79]. All the above demographic and resource utilisation attributes have been demonstrated in *B. dorsalis* competing against other species.

## 3. Proposed Scenarios and Biosecurity Impacts

Adaptive biosecurity governance acknowledges uncertainties [80] and therefore scenarios are not only appropriate, but necessary, to explore potential risk [42]. We recognise three outcomes of fruit fly competition which have the potential to impact the biosecurity risks associated with a successful fruit fly invasion, and a fourth impact which need not be linked to invasion but where competition may nevertheless influence the outcome of a plant health intervention. These are:

- Replacement or reduction of a previous key pest following establishment of a new pest,
- Displacement of existing pest species in space, time or host following establishment of a new pest,
- Non-establishment of a new pest in areas with competitively dominant species,
- Lesser fly resurgence following removal of a competitively superior species.

Within the first of these four impacts, we recognise three different biosecurity implications, making six different competition mediated biosecurity effects overall. Each of these is introduced below, and then illustrated by scenarios relevant to Australia/New Zealand and Europe. All our scenarios deal with cases where two or more fruit fly species could interact. We note that including competition into a fruit fly PRA is irrelevant if only one species is involved, e.g., a PRA for an exotic species where the country/region of concern has no existing fruit fly species. Additionally, competition is likely to be of lesser importance if the host usage of potentially competing fruit fly species has little or no overlap. For example, in La Reunion, *B. dorsalis* has had only weak competitive effects against cucurbit specialist tephritid species, as cucurbits are infrequently used by *B. dorsalis* [40].

### 3.1. Replacement or Reduction of a Previous Key Pest following Establishment of a New Pest

Biosecurity implication 1: Change in pest management recommendations/practice for previous pest species

While it is self-evident that the introduction of a new pest into an area will require new management research and recommendations, what may be less obvious is the need and cost involved with changing management practices for existing pests. Such changes may be relatively trivial; for example, in Tahiti, the switch of the dominant pest from Queensland fruit fly to Oriental fruit fly [55] requires only the addition of a new lure type in male annihilation devices (using methyl eugenol as well as cue-lure) for all existing and new control recommendations to remain current. Or there may be no impact if the controls developed for the existing pest are immediately transferable to the new pest. However, the impacts of changing practice could be much more significant if major investments in the previous control strategies are not easily translatable.

***Scenario.*** Should Oriental fruit fly invade eastern Australia, as it did in the 1990s prior to eradication [81], then available evidence suggests that it would rapidly displace Queensland fruit fly as the dominant fruit fly pest [55,82]. In the last decade approximately AUD$50 million has been spent on the research and infrastructure needed to implement an operational Sterile Insect Technique (SIT) program against the Queensland fruit fly in Australia (https://www.horticulture.com.au/hort-innovation/our-work/hort-frontiers-strategic-partnership-initiative/fruit-fly-fund/ (accessed on 7 November 2022)). An Oriental fruit fly invasion would potentially make the SIT investment in Queensland fruit fly control largely redundant in all except the coldest parts of the country (see Australasian scenario Section 3.2) and so represent lost revenue [44].

A risk assessment for Oriental fruit fly for Australia should therefore include the cost of the “lost research” of the Queensland fruit fly SIT program, and the new research and infrastructure costs of repurposing the Queensland fruit fly mass-rearing facility for handling Oriental fruit fly if SIT was considered an option for that species.

Biosecurity implication 2: Displacement of existing species may indirectly benefit pest management

The displacement of at least one, sometimes several pest species by sequentially superior invasive species (see, e.g., [35,40,59]) may have indirect benefits for pest management practice. If the invasive fly is displacing multiple existing species, then the ability to focus controls on just the one dominant species may be more efficient for researchers, extension staff and growers than having to use a suite of different controls for different species. This may particularly be the case for an invasive *Bactrocera* species, as their response to male lures make the control of these flies through the male annihilation technique highly efficient [83,84].

We recognise this scenario, of suggesting a biological invasion may have potential benefits, is controversial and it is not meant in any way as a justification for lessening quarantine or emergency response efforts. Indirect gains in pest management efficiency are largely meaningless if the new pest causes significantly more crop damage than the displaced pest(s) (see, e.g., [85]). However, where two pest species (or one new species and an existing community of species) are both equally damaging, and the invasive species is easier to control, then for the economic analysis component of a PRA the negative costs of the new invasive species may be partially offset by indirect gains in pest management efficiency and a reduction of the impacts of the less competitive species.

***Scenario.*** Oriental fruit fly, Queensland fruit fly and Mediterranean fruit fly are all highly invasive, polyphagous fruit flies. All are absent from New Zealand, a country with an extensive horticultural industry, and for that reason they are priority biosecurity pests (https://www.mpi.govt.nz/biosecurity/priority-pests-diseases/ (accessed on 7 November 2022)). While these pest species are not wanted, a PRA for Oriental fruit fly could look quite different if the other flies were absent, or already established in the country. In the absence of other species an invasion of Oriental fruit fly is nothing but bad, causing only damage. However, if Mediterranean fruit fly were already established, for example, the negative aspects of an invasion by Oriental fruit fly would be partially offset by the indirect benefits of reducing Mediterranean fruit fly impacts and, because of the strong methyl eugenol response [86], Oriental fruit fly field control may be easier to implement and more effective than Mediterranean fruit fly control. However, nevertheless, we would not predict Oriental fruit fly to entirely eradicate Mediterranean fruit fly, merely displace it in crop, time, or location, so ultimately any invasion is to be avoided.

Biosecurity implication 3: Change in market access status with establishment of a new pest.

Competitive displacement of a previous key pest by a new pest of quarantine concern has immediate and obvious negative market access consequences, as market access protocols need to be developed and negotiated for the new pest [87]. However, the market access implications for the now lesser pest(s) may also require renegotiation. The level of competitive displacement may be such that the displaced pest(s) are no longer a biosecurity risk because they are now rare or absent from the crop (see, e.g., the reduced commercial host list of peach fruit fly after displacement by Oriental fruit fly in La Reunion [40]). Depending on the new situation, and negotiation with the trading partner(s), the old pest may no longer be a pest of market access concern, or it may (and most likely will) remain of a pest of market access concern even if to producers it is no longer a priority issue for in-field management. This issue becomes increasingly complex when there are multiple pest species present in the invaded area.

***Scenario.*** The Australian state of Western Australia (WA) is recognised by Australian domestic and some international trading partners as containing only one fruit fly species of market access concern, Mediterranean fruit fly. If Queensland fruit fly invaded Western Australia from eastern Australia, it is likely that Queensland fruit fly would largely displace Mediterranean fruit fly in some production areas or crops [70], although full extinction of Mediterranean fruit fly from WA is unlikely [26]. In the worst-case trade scenario, WA now has two major pest flies of market access concern for which all exported commodities would need phytosanitary treatment. In a best-case scenario, Mediterranean fruit fly is no longer of biosecurity concern having been replaced by Queensland fruit fly and benefits in harmonisation of export protocols would be accrued nationally (realistically this is highly unlikely under most market access negotiations). A mid-case scenario is that Mediterranean fruit fly could be managed for export through new or modified protocols if cheaper to implement than existing protocols (e.g., utilising an Area of Low Pest Prevalence). A PRA for the entry of Queensland fruit fly into WA thus needs to consider the range of scenarios.

### 3.2. Displacement of Existing Species in Space, Time, or Host with Establishment of New Pest

Biosecurity implication 4: Previously existing fruit fly pests are still a problem, but in a different location, season, or host plant

With the exception of the inferential case of Mediterranean fruit fly in eastern Australia ([70,71] and see Supporting Document S1), invasive fruit flies are not known to have driven other fruit fly species to extinction, except, and again inferentially, on islands with already rare endemic species [31]. Rather, the more common pattern seen is that displaced species may be reduced in numbers but persist by utilising hosts less used by the new species [58,88], using locations in the landscape less preferred by the invasive species (e.g., higher altitude or dryer areas) [40,57,89], or by being temporally asynchronous with the invasive species [66,67].

***Scenario***. Queensland fruit fly is a highly polyphagous species [90], but in the field some crops are only infrequently attacked [91,92]. In Tahiti, Oriental fruit fly proved to be competitively superior to Queensland fruit fly and displaced it from crops such as mango and guava [55]. In far-northern Australia Oriental fruit fly also proved competitively superior to Queensland fruit fly during the 1990s incursion [82]. Should Oriental fruit fly re-enter and establish in Australia then it is likely that Queensland fruit fly may disappear as a pest of many crops, replaced by Oriental fruit fly, but currently low-pressure Queensland fruit fly crops may become much more heavily utilised by that fly. This indirect effect would make the impact of an Oriental fruit fly incursion greater than the direct effects of Oriental fruit fly damage alone. Queensland fruit fly may also be competitively “pushed” to dominate in southern Australia, while Oriental fruit fly dominates in tropical and subtropical Australia because of the greater cold tolerance of the former species [18] over the latter [93].

### 3.3. Non-Establishment of a New Pest in Areas with Competitively Dominant Species

Biosecurity implication 5: In regions where a competitively superior species is already established, the likely establishment of an invasive but competitively inferior species is very low.

In their review of fruit fly competition, Duyck et al. [26] note a corollary of competitive displacement, which is competitive resistance. They noted, and there have been no contrary observations since, that there are no records of fruit fly species invading and persisting in a region or country where an already more competitive species is established. Thus, while *Bactrocera* species have invaded regions previously dominated by *Ceratitis* or *Anastrepha* species, and *Ceratitis* have invaded *Anastrepha* regions, there are no reverse examples. That is neither *Anastrepha* nor *Ceratitis* have invaded areas dominated by *Bactrocera*, nor has an *Anastrepha* species invaded an area dominated by *Ceratitis*. In the invasion biology literature this is known as ecological resistance and is commonly attributed to competitive interactions against the invading species by already present species [94,95]. Propagule pressure can overcome ecological resistance [96], but given the generally accepted large sizes of endemic pest fruit fly populations [97,98], and the well-regulated movement of fruit to minimise propagule pressure [4,5], the chances of a less competitive fruit fly species establishing in an area where a more competitive species is already endemic appears unlikely.

***Scenario***. The Australian “east–west” divide is based on Mediterranean fruit fly being established in Western Australia (WA), Queensland fruit fly being established in the Australian east-coast states of Queensland, New South Wales, and Victoria, and neither species being established in the state of South Australia, which sits between WA and the eastern seaboard. While there is biological potential for Queensland fruit fly to invade WA, as Queensland fruit fly is competitively superior to Mediterranean fruit fly (Duyck et al. 2004), the probability of the reciprocal invasion of Mediterranean fruit fly into the east coast should be considered very low for the same reason. This conclusion was also drawn by Dominiak and Daniels [70] and Dominiak and Mapson [71].

### 3.4. Lesser Fly Resurgence following Removal of a Competitively Superior Species

Biosecurity implication 6: That removal of a competitively dominant species through a species-specific control strategy (such as MAT or SIT) will result in a previously minor pest becoming a major pest.

While not directly related to the PRA process, which generally refers to forward planning for the invasion/entry of an exotic pest, this last scenario is still pertinent to those charged with plant-health planning although in this case dealing with the control of an already established endemic or exotic species.

Pesticide-induced pest resurgence is a phenomenon in which the removal of a dominant pest with a targeted pesticide sees a minor pest released from a previously suppressed state to become a new major pest [99,100,101]. Bateman [24] suggested a similar situation could arise with fruit flies if a competitively superior species was removed from a system where two or more competing pest fruit flies occurred. Biasazin et al. [39] have documented this exact effect in Ethiopian orchards. Invasion by Oriental fruit fly initially displaced the previously dominant Mediterranean fruit fly. However, when Oriental fruit fly populations were suppressed using the species-specific male annihilation technique, Mediterranean fruit fly numbers resurged. Ito [102] reported the same effect on Japanese islands of the Ryukyu Archipelago, with *Z. cucurbitae* populations gradually increasing over time after the eradication of *B. dorsalis*. The likely resurgence of lesser flies needs to be considered if planning any new control initiative which selectively targets only one pest species, be that species a newly invasive fly (as in the case of the Ethiopian example), or an endemic.

***Scenario I***. The Sterile Insect Technique is a logistically expensive and managerial intensive control technique [103,104]. By the nature of the method involved, i.e., interrupting the mating of wild flies through release of sterilised males of the same species [105], it is also 100% species specific. In tropical and warm temperate Australia, Queensland fruit fly is numerically dominant over “lesser” pest species, such as Lesser Queensland fruit fly (*B. neohumeralis*) [106], possibly due to competitive advantage [107,108,109]. If SIT, or another species-specific control approach, was successfully applied against Queensland fruit fly, then it is possible that one of the currently competitively inferior species would emerge as the new major pest.

***Scenario II*.** The argument is made above (Section 3.3) that the presence of *B. tryoni* is likely to exclude *C. capitata* from invading eastern Australia through biotic resistance. *Bactrocera tryoni* may also exclude previously tropical species such as *B. neohumeralis* from invading Victoria from the north, as climate change makes Victoria climatically suitable for these species [17]. However, if *B. tryoni* populations were greatly reduced through the application of SIT, then this biotic resistance would be removed/reduced and the likelihood of invasion of Victoria by a second fruit fly species is greatly increased.

## 4. Conclusions and Recommendations

The entry of a new pest fruit fly into a region will always have negative consequences because of direct impacts on crop production and market access. This effect is most dramatic, and economically damaging, in areas previously free of a pest fly [110]. However, where an invasive species is establishing in a region where one or more pest fruit fly species already occur, the biological and economic impact of the new species will be modified, for better or worse, because of competitive interactions between species. By displacing existing pest species into new crops, or areas previously at low risk from resident species, the negative effects of the new invasion may be amplified. Alternatively, if competition sees the simple within-crop replacement of one pest with another pest, then the economic and biological impacts may be closer to being neutral. The displacement of an existing species by a new species will also impact on previous investments in pest management research and infrastructure and may impact on the negotiated trade agreements for the previously established species. The observation that an invasive fly may have indirect benefits by reducing other pests should never be used as an argument to ease biosecurity arrangements for preventing invasions, but we do argue that the economic costings component of a PRA should recognise that a fruit fly invasion, however much unwanted, may have indirect beneficial impacts if competitive displacement of an existing fruit fly pest is likely.

As demonstrated through the scenarios there is no simple good/bad, plus/minus pattern in how fruit fly competition may impact on the pest risk assessment, but there is ample evidence that competitive effects should be factored into fruit fly PRAs while recognising that it adds a layer of complexity to any subsequent modelling [111]. We thus strongly recommend that when writing PRAs for tephritid species, or before beginning large scale control initiatives, how competition between tephritids may modify risk or benefit be included in assessments. For an individual country or region the impact of an invasive fly’s potential competitive interactions with existing species will be unique to that country or region due to local farming practices and purpose (e.g., local consumption versus export), environmental conditions, the impact of existing pests, etc. For this reason, as it is for any part of the PRA or pest management process, local conditions need to be considered. The scenarios we use above to illustrate the biosecurity implications of competition are pertinent only to Australia and New Zealand at the current time and will not be relevant to other countries, or to Australia and New Zealand under future conditions.

We also finish by noting that the tephritid competition literature is dominated by work on just a handful of species and that the ability to extend competitive interactions more broadly into fruit fly PRAs needs experimental work done on a greater range of genera and species, for example *Rhagoletis* spp. and *Drosophila suzukii* (Matsumura). Further work may demonstrate that currently ‘generalised’ patterns of competitive interactions (e.g., of competitive hierarchies or displacement effects) may not, in fact, be general at all and are rather an artifact of the relatively small number of species so far studied. Thus, for potentially high-impact interactions, for example how *B. dorsalis* would interact with *B. tryoni* in Australia if the former species entered and established, targeted research on the particular species pair is recommended so that any assumptions made within PRAs are based on specific data, rather than assumptions made based on studies with other species in other agricultural systems. Notably, many more studies on the processes of tephritid competition are needed (e.g., [29,74]), rather than relying on changing patterns of abundance or host use to infer the processes of tephritid competition, as ecological processes tend to be much more consistent across time and space than do patterns [25,112] which is critical if they are to be incorporated into PRAs predicting future events.

## Data Availability

No new data was created for this paper. All sources of information are fully cited and are in the public domain.

## Supplementary Materials

The following supporting information can be downloaded at: https://www.mdpi.com/article/10.3390/insects13111065/s1. This paper has one Supporting Document, a mini-review summarising what is known about the putative competition which occurred between Queensland fruit fly (*Bactrocera tryoni*) and Mediterranean fruit fly (*Ceratitis capitata*) in eastern Australia in the years between ~1900 to 1940. [113,114,115,116,117,118,119,120] are cited.
