# Supplementary material for: Competition: A Missing Component of Fruit Fly (Diptera: Tephritidae) Risk Assessment and Planning
Source: Insects. 2022 Nov 17;13(11):1065. doi: 10.3390/insects13111065 (PMC9697728; doi:10.3390/insects13111065)
Supplement: Supplementary file 1 [file insects-13-01065-s001.zip › insects-2007767-supplementary.pdf]

Supporting Document S1

Evidence for *Bactrocera tryoni* driving *Ceratitis capitata* to extinction in Eastern Australia in the 1930s

Background

Prior to World War II, Mediterranean fruit fly (Medfly) was established on the Australian east coast, most likely arriving there from Western Australia where it had entered in the late 1800s [113]. Indirect evidence (frequency of reports in agricultural journals, statements in grower leaflets, etc) suggests that Medfly was more abundant than Queensland fruit fly (Qfly) in the first decades of the 1900s in NSW and Victoria. But over time Medfly became rarer in eastern Australia, such that it has been absent from the eastern states since the beginning of the 1940s [70,71].

Queensland fruit fly drove Mediterranean fruit fly to extinction

This disappearance of Medfly, and the subsequent increased importance of Qfly, is routinely interpreted as Qfly out-competing Medfly to the point of extinction [26,70,71,114,115]. So frequently is this assertion repeated that it is not unreasonable to assume that this interpretation is supported by hard data; unfortunately, this is not the case.

Suggestions that the extinction of Medfly in eastern Australia was driven through competitive exclusion by Qfly are speculative, based on no contemporary or modern data. International literature strongly suggests that Qfly, as a *Bactrocera* species, should be competitively superior to Medfly, a *Ceratitis* species [26], but no competition experiments between this pair of species have ever been done. The only Australian study from when the species co-occurred in the field suggests that Qfly may have been competitively dominant, with Allman [116] reporting (with no data) that Qfly females were more aggressive than Medfly females and (with data) that when co-infesting apples more Qfly than Medfly adults emerged (data from 9 fruits) and the

emergent Medfly adults were very small. However, he also presented data showing that Medfly did better in pears than Qfly (data from 3 fruits). Other than Allman there is no contemporary or modern documentation about interactions between Medfly and Qfly. All subsequent statements about Qfly driving Medfly to extinction through competition are speculation, re-citation, or miss-citation. As an example, Dominiak and Daniels [70] make the statement "*Numerous authors have suggested that one of the major reasons Qfly displaced Medfly was because Qfly laid eggs in Medfly oviposition holes (Anon 1939, Allman 1939, O'Loughlin 1975, Hely et al. 1982)*". Normally this would suggest that four separate studies had each presented original data to support the conclusion. Study of the papers show that this is not the case. Allman [116] makes the original observation (unsupported by data) that Qfly will oviposit into previous Medfly oviposition sites; all subsequent sources then simply repeat that information. Importantly, not all of the subsequent authors even seem to believe it. For example, rather than being a supporter of the oviposition theory as the cause of displacement as inferred by Dominiak and Daniels [70], O'Loughlin [117] actually says: "*It has been suggested that Queensland fruit fly succeeded in displacing Medfly because of its habit of utilising for its own egg-laying purposes, the oviposition punctures made by the Medfly. It remains difficult to believe that there was not room for both species especially as the Queensland fruit fly co-exists in some places with other related species*." Other authors have also been critical of the idea that the fly's oviposition behaviour was a mechanism by which Qfly competitively displaced Medfly [25,118].

As the only contemporary source to have noted the decline of Medfly in eastern Australia, it is important to read Allman [116] closely. In addition to "antagonism" between the species, he also talks about targeted crop hygiene and the introduction of new pesticide sprays which "*have now come into general use*". He also notes that Medfly was more abundant in some districts than others, and was occasionally collected in different seasons to Qfly, which hints at temporal

and geographic displacement of Medfly, rather than competition leading to extinction. With respect to Qfly becoming more common than Medfly he records that "*The statement is, therefore, often made that the Queensland fly, has, during the past 30 years, displaced the other species*", but he does not at any point say that he endorses this view; he is simply noting it as something which is said. In contrast, he does explicitly state: "*Factors which have possibly contributed to a lessening importance of the Mediterranean fly, especially in the County of Cumberland, may be the enforcement of regulations regarding the picking up and destruction of infested fruit, together with the enforced removal from trees of certain varieties of citrus fruit, e.g. Seville oranges and mandarins, which seem to be preferred hosts for this species*."

All data from related international studies, along with the observations and very small amount of data presented in Allman [116], predicts that Qfly will be competitively superior to Medfly if formally tested; this issue is not debated. What is debated is an assumption that competition from Qfly was solely responsible for the extinction of Medfly from eastern Australian. The extinction of a fruit fly species through competition with another fruit fly has not been documented anywhere in the world, although Mze Hassani et al. [31] *speculate* it may have occurred for two already rare endemic species on an Indian Ocean island. Rather than extinction, competitively inferior fruit fly species are normally displaced in host, space or time by a competitively superior species [26]. The disappearance of Medfly from eastern Australia may certainly have been influenced by competition with Qfly, but equally the advent of the widespread pesticide applications and targeted crop hygiene controls [116], outbreak intervention [113], seasons which may not have suited an insect adapted to a Mediterranean climate [118,119], and an allee effect coming into play once the population had shrunk [120], could all have added to the eventual extinction of Medfly.
